# Chatbot-Based Version of a World Health Organization–Validated Intervention for Stress Management in Patients With Breast Cancer (Self-Help Plus): Protocol for a Pilot Feasibility Study

**DOI:** 10.2196/65837

**Published:** 2025-08-26

**Authors:** Valentina Fietta, Silvia Rizzi, Lorenzo Gios, Silvana Selmi, Chiara De Luca, Lucia Pederiva, Stefania Poggianella, Maria Chiara Pavesi, Monica Campregher, Silvia Lazzeri, Sara Cantarelli, Marianna Purgato, Corrado Barbui, Nicolò Navarin, Silvia Gabrielli, Merylin Monaro, Stefano Forti, Antonella Ferro

**Affiliations:** 1 Department of General Psychology University of Padova Padova Italy; 2 Digital Health Research, Centre for Digital Health & Wellbeing Fondazione Bruno Kessler Trento Italy; 3 TrentinoSalute4.0 - Competence Center for Digital Health, Province of Trento Fondazione Bruno Kessler Trento Italy; 4 Psychology of Palliative Care, Pediatric Palliative Care, and Pain Management National Council of the Order of Psychologists (CNOP) Roma Italy; 5 Istituto Pavoniano Artigianelli Trento Italy; 6 Breast Unit Healthcare Trust of the Autonomous Province of Trento (APSS) Trento Italy; 7 WHO Collaborating Centre for Research and Training in Mental Health and Service Evaluation Department of Neurosciences, Biomedicine and Movement Sciences, Section of Psychiatry University of Verona Verona Italy; 8 Department of Mathematics Tullio Levi-Civita University of Padova Padova Italy

**Keywords:** stress management, psychological well-being, breast cancer, eHealth, mobile health, mHealth, mobile apps, development, usability, user-centered design, artificial intelligence, AI

## Abstract

**Background:**

Emerging digital tools play an innovative and key role in supporting women’s psychological well-being throughout the different stages and challenges of cancer. The development and adoption of digital interventions, including chatbots and virtual coaches within smartphone apps, are increasingly recognized as valuable resources for enhancing women’s mental health.

**Objective:**

The aim of this paper is to present the research protocol for a pilot study designed as a proof-of-concept investigation. The study evaluates the feasibility, acceptability, and perceived utility of a mobile app delivering an acceptance and commitment therapy–based stress management intervention. The intervention is delivered through ALBA (A Well-Being Assistant), a virtual coach embedded within the TreC (an acronym for *cartella clinica del cittadino*, meaning “citizen’s electronic health record”) research platform—a mobile health ecosystem designed to support research and digital health interventions. ALBA guides users through 5 coaching sessions tailored for women undergoing breast cancer (BC) treatment. The chatbot-delivered app is an adaptation of Self-Help Plus, a World Health Organization (WHO)–validated stress management intervention, and is provided in text, audio, and video formats. The intervention’s potential impact on participants’ psychological well-being is also explored.

**Methods:**

A convenience sample size of 50 participants will be identified to meet the study’s objectives. Participants will be recruited using a convenience sampling approach from women receiving care at the Breast Unit of the Azienda Provinciale per Servizi Sanitari di Trento. ALBA will interact with the participants for 6 weeks. Specifically, there will be 1 coaching session per week, followed by weekly assigned acceptance and commitment therapy exercises to be performed between sessions.

**Results:**

The app is expected to demonstrate high usability and engagement, aligning with the WHO Self-Help Plus protocol. Improvements in psychological well-being and quality of life are anticipated. Data from this pilot will be analyzed using both quantitative and qualitative methods, with a focus on assessing feasibility, acceptability, and perceived utility and usability in supporting women during BC treatment.

**Conclusions:**

Existing literature indicates a promising role for new technologies in delivering validated mental health interventions, highlighting the potential of digital interventions to address barriers related to social stigma and seeking assistance. This pilot is expected to provide valuable insights on the potential acceptability and usefulness of providing consistent mobile health psychoeducational support to women throughout the course of BC.

**International Registered Report Identifier (IRRID):**

PRR1-10.2196/65837

## Introduction

### Overview

A growing need for psychological support is emerging among the general population, and this need increases even more if we consider populations exposed to stressful situations [1,2]. Emerging scientific literature highlights that psychological vulnerability is often linked to specific periods along the disease trajectory, such as those experienced by women with breast cancer (BC). On the one hand, psychological distress, including stress, anxiety, and depression, often accompanies pathologies such as BC [3]. On the other hand, having a more positive attitude contributes to disease management and recovery, highlighting the importance of addressing mental health issues on physical aspects while taking charge [4,5]. Therefore, improving the mental well-being of women dealing with BC could be an effective way to enhance their overall health. In this sense, evidence of the effectiveness of psychological interventions aimed at women with BC is increasing [6,7], but access to support services still presents several challenges. Many women face geographical barriers, with specialist centers often located far from their homes. Furthermore, the shortage of qualified personnel, such as psychologists, limits access to specialist care. The stigma associated with mental health problems can also prevent women from seeking psychological support in these circumstances [8,9].

Within this framework, there is a recent focus on the potential contributions of digital technologies in fostering psychological well-being, recognizing their capability to provide an optimal solution to reduce barriers [10] and enhance patient empowerment [11].

Indeed, the World Health Organization (WHO) has implemented a comprehensive mental health strategy grounded in principles of inclusivity and scalability, which also incorporates the use of digital technologies [12,13]. The study described in this protocol is part of the panorama of WHO’s strategies, and it aims to evaluate the acceptability and feasibility of an intervention delivered via digital tools to promote psychological well-being. In particular, this intervention, initially validated by WHO itself and called Self-Help Plus (SH+) [14-16], has never been used on the target population of this study, women with BC. SH+ is a low-intensity and transdiagnostic intervention that is easily adaptable in different contexts, delivered by nonspecialist operators, and has solid evidence-based psychological principles inserted in a self-help approach. This approach has already been validated with various populations considered vulnerable, such as migrants, refugees, and health care workers, both to promote psychological well-being and for clinical intervention purposes, demonstrating general acceptability and potential effectiveness [16,17]. The first attempt was made to make the protocol web based within the RESPOND (preparedness of health systems to reduce mental health and psychosocial concerns resulting from the COVID-19 pandemic) project, but the involvement of a human person, in the role of a helper, was still necessary [18].

Cognitive behavioral therapy (CBT) techniques, and in particular those referring to third-generation therapy, are shown to be more suitable than other psychotherapeutic approaches to be transferred into low-intensity interventions delivered by nontraditional methods such as eHealth applications [19]. SH+ is based on the principles of acceptance and commitment therapy (ACT), a third-wave form of CBT [20,21], and is divided into 5 chapters that address 5 strategies for managing stress and promoting psychological well-being. The five chapters, later translated into sessions, are (1) grounding (mindfulness), (2) unhooking (defusion), (3) acting on your values (values-based behavioral activation), (4) being kind (gratitude), and (5) making room (acceptance).

### This Study

#### Overview

Our study is placed within the design and development cycle of the Obesity-Related Behavioral Intervention Trials (ORBIT) model [22], specifically in phase 2a, “refine.” It involves providing material based on SH+ intervention in text, audio, and video format by a virtual coach (ie, a digital chatbot assistant, identified as ALBA [A Well-Being Assistant]) implemented within the TreC (an acronym for *cartella clinica del cittadino*, meaning “citizen’s electronic health record”) research app (a platform or app purposively designed to enable the delivery of several types of mobile health [mHealth] features). It could be stated that this study is innovative for 2 main reasons. First, it involves the digitalization of an intervention that was originally delivered in person and in group settings [14,15]. The ALBA chatbot version is designed to be more accessible and tailored to the individual, functioning without the presence of a helper, unlike in the RESPOND project [17,18], and includes chatbot-guided activity completion between sessions, which differs from the group session delivery format. Therefore, it is essential to evaluate how effectively this new format achieves these goals. Second, this study introduces a novel focus on a population consisting exclusively of female individuals and those considered medically vulnerable, which represents a significant new factor in evaluating the feasibility, acceptability, and efficacy outcomes. The proof-of-concept (POC) study, described in this research protocol, aims to test both the transferability and acceptability of the digitalized intervention from the original, context-specific in-person format (refer to the Primary Objective section), as well as to assess its initial efficacy on the targeted psychological variables in this newly defined population (refer to the Secondary Objective section).

#### ALBA Intervention

ALBA is an intervention based on ACT techniques; it aims to promote the quality of life and enhance stress-coping skills in women with BC through a psychoeducational approach. The module is self-administered and available for both Android (Google LLC) and iOS (Apple Inc) devices. All the contents of the dialogues were developed by a group of researchers belonging to the Digital Health Research of the Bruno Kessler Foundation of Trento and psychologists belonging to the Pavoniano Artigianelli Institute with specific skills in the communication field starting from the SH+ manual [23] and subsequently reviewed by a psychologist manager of the psychology operational unit of the Provincial Health Services Authority of Trento (Azienda Provinciale per i Servizi Sanitari di Trento). The dialogues, videos, and audio tracks were developed according to educational logic. Furthermore, the structuring of the material and the delivery methods were supervised by professionals from the WHO Center for Research in Mental Health of the University of Verona, who have already carried out implementation studies and validation of SH+ at national and international levels.

To ensure the best possible adaptation of the intervention to our target sample, we previously conducted a co-design study based on guidelines of a previous step of the ORBIT model [22]. This process involved domain experts, such as psychologists, SH+ experts, and communication and usability experts, and a small sample of women with BC and oncological clinicians. For further details, please refer to our previous study [24].

#### Technological Tools

This intervention is part of a behavior change framework [25] aimed at supporting women’s psychological well-being. The term “intervention” refers to the virtual coach, the chatbot ALBA, which delivers structured, rule-based informational content reviewed by psychological professionals. ALBA functions as a tool for information delivery, not as a conversational agent, as it does not engage in real-time, dynamic interactions. While it collects and transmits data, it is primarily a chatbot for eHealth interventions. ALBA is developed for research purposes to validate the digital health version of SH+ focused on ACT practices for women with BC.

ALBA, the technological component of this study, is built upon the general TreC platform, a public mHealth platform enabling residents of the Autonomous Province of Trento (North Italy) to access, manage, and share their health and well-being information. TreC is a robust and reliable platform designed as a “system of systems” rather than merely a data repository [26]. Indeed, its flexible architecture allows for collecting and managing diverse data types and supports developing and integrating additional subsystems to offer specific functions. A key feature of TreC is empowering users to manage their health-related data, functioning as a personal health record.

**Figure 1:**
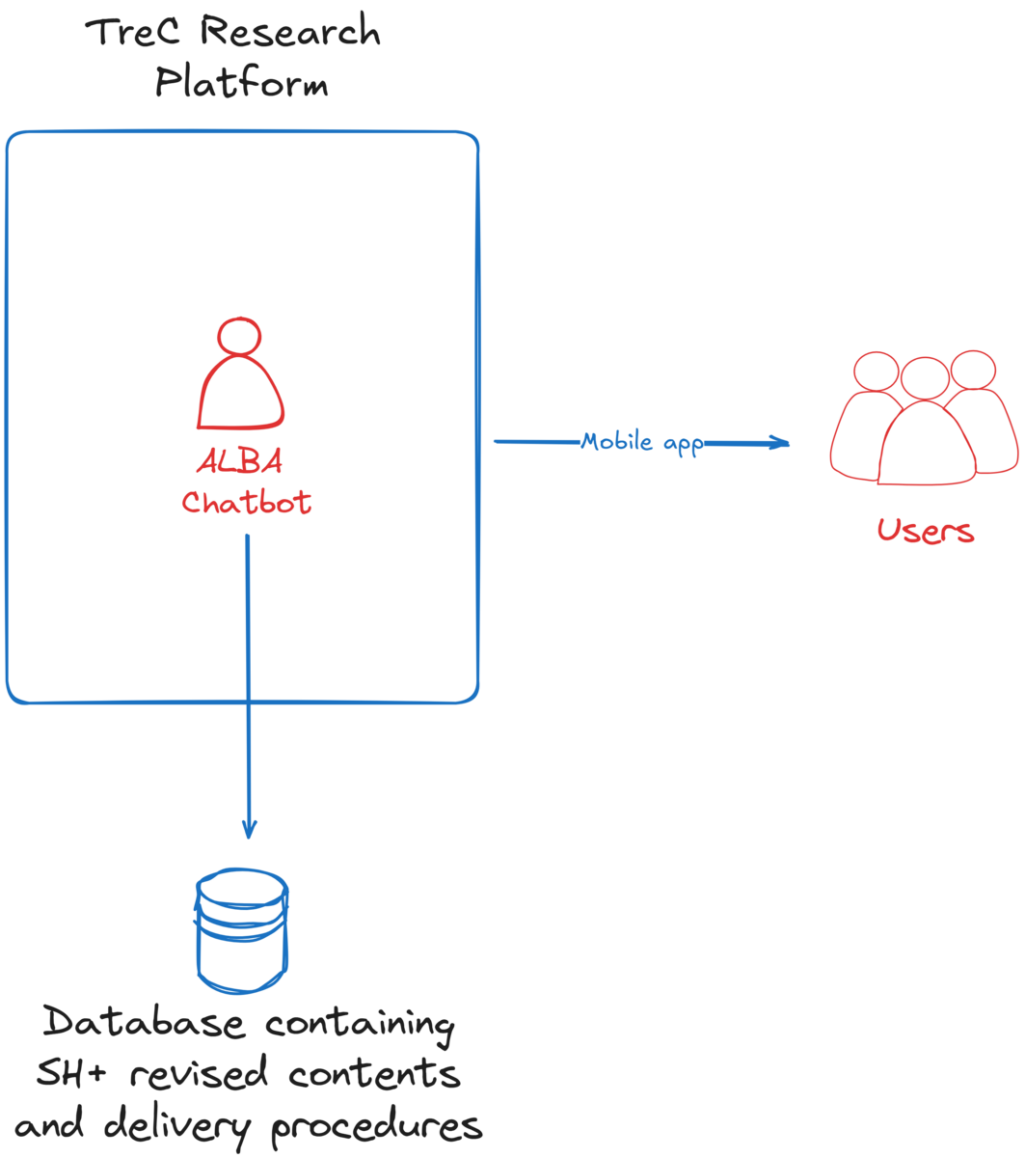
Graphical representation of the TreC (an acronym for cartella clinica del cittadino, meaning “citizen’s electronic health record”) research platform, which incorporates the ALBA (A Well-Being Assistant) chatbot and illustrates its interactions with the database and users. SH+: Self-Help Plus.

Within this general platform, a specific mHealth ecosystem has been designed to support research and digital health interventions, called TreC research (“Trec Ricerca” in Italian; Figure 1). This module is available for free download as a mobile app to all women diagnosed with BC. Authentication is required via the secure, General Data Protection Regulation–compliant national identification system (Sistema Pubblico di Identità Digitale).

#### SH+ Sessions

**Figure 2:**
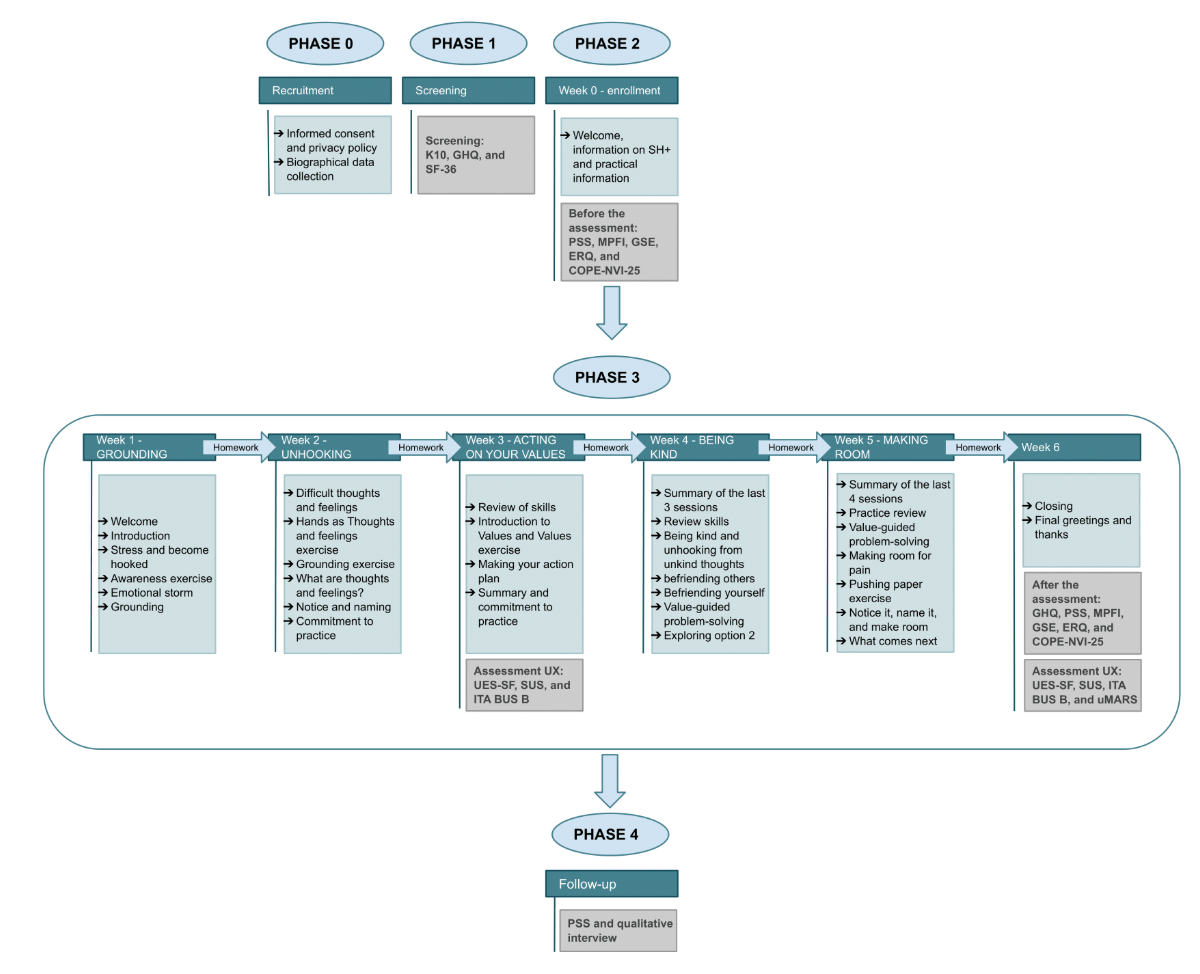
Graphical representation of the conversational protocol delivered to users and its chronological structure. COPE-NVI-25: Coping Orientation to the Problems Experienced; ERQ: Emotion Regulation Questionnaire; GHQ: General Health Questionnaire; GSE: General Self-Efficacy Scale; ITA BUS B: Italian Chatbot Usability Scale, version B; K-10: Kessler Psychological Distress Scale; MPFI: Multidimensional Psychological Flexibility Inventory; PSS: Perceived Stress Scale; SF-36: Short Form Health Survey 36; SUS: System Usability Scale; UES-SF: User Engagement Scale-Short Form; uMARS: User Mobile Application Rating Scale.

The intervention spans a total duration of 6 weeks. For each week, a session of approximately 40 minutes is delivered, while this can be divided into two 20-minute subsessions according to users’ preferences. Phase 3, represented in Figure 2, provides a graphical depiction of the conversational protocol delivered to the participants and its chronological structure. The intervention’s contents are presented through a gradual process designed to progressively increase the participant’s self-awareness and mindfulness-oriented skills, including accepting and normalizing internal states, managing stress, and enhancing psychological well-being. Participants are also required to complete tasks and fill in a dedicated e-diary. The program is structured into 5 main coaching sessions, following the operational chapters of the SH+ manual, each addressing a specific strategy for managing stress and promoting psychological well-being, which are the main aims of the intervention. These 5 modules, translated into weekly e-sessions, are grounding (mindfulness), unhooking (defusion), acting on your values (values-based behavioral activation), being kind (gratitude), and making room (acceptance; phase 3 in Figure 2).

### Goal of the Study and Research Questions

#### Overview

Therefore, this protocol paper aims to describe the POC developed to deploy the ALBA app to women with a BC diagnosis. The POC aims to evaluate the acceptability and feasibility of delivering SH+ through ALBA, a platform designed to provide ACT-based psychoeducational sessions. ALBA supports users in completing exercises and tasks assigned between coaching sessions and periodically delivers structured information—curated by professional psychologists—in audio, text, and video formats. This material is independently accessed and managed by users. The virtual coach serves as an information transmission tool, while the app also administers questionnaires and manages data collection.

This behavioral change intervention [25] aims to empower women with adaptive strategies for managing stress. Studying its feasibility can represent a first step to evaluate the possibility of including a digital service to support and promote psychological well-being in the context of stepped care for women in the BC pathway.

#### Primary Objective

The main objective of the POC study is to evaluate how women experience the chatbot-based version of the WHO-validated intervention SH+. It aims to assess the following areas: (1) the user experience (UX) and the usability of the virtual coach, ALBA, installed in the TreC research app environment and (2) how users felt during the intervention, using ALBA, as reported through semistructured interviews. The IT evaluation of the TreC research app itself will be presented in another study.

#### Secondary Objective

The secondary goal is to assess the efficacy of the intervention on women’s psychological well-being through the administration of self-report pretest-posttest and follow-up questionnaires.

## Methods

### Design and Study Plan

The study is articulated in 4 main phases depicted in Figure 2 and extensively described in the Data Collection section.

#### Phase 0: Recruitment

This phase will take place at the hospital’s Breast Unit, and it will be carried out by the health professionals of the Breast Unit (physicians and case manager nurses).

#### Phase 1: Screening

The screening phase is carried out by psychologists, and subsequent included participants are given access to the TreC research app.

#### Phase 2: Enrollment at Week 0

At the beginning of the path, ALBA will administer 5 self-report questionnaires to establish the baseline level of the examined variables (preassessment questionnaires). The participant can choose the best day and time to interact with ALBA (first interaction with ALBA).

#### Phase 3: Intervention Delivery From Week 1 to Week 5

Specifically, there is 1 psychoeducational session per week, and the contents will be presented in different formats (text, images, audio, and video tracks). Moreover, ALBA provides users with instructions on how to carry out simple exercises and fill in a personal diary. The questionnaires will be further administered by ALBA at the end of the course (at week 6) to verify whether participants have perceived a change in terms of psychological well-being and quality of life. In total, 4 questionnaires will be administered to evaluate usability and UX (weeks 3 and 6).

#### Phase 4: Follow-Up

Four months after the conclusion of exposure to SH+ intervention, women who have given consent will be invited to participate in a semistructured interview to investigate their experience of using ALBA and the TreC research app to understand how the women felt during the intervention. In addition, the long-term effects of stress will be collected by administering a follow-up questionnaire.

### Participant Recruitment and Withdrawal

#### Overview

The study will take place in the Province of Trento (North Italy), where approximately 5000 women have or have had a diagnosis of BC, with almost 500 new cases every year [27]. In January 2018, the Breast Unit was established in the Province of Trento. It represents an innovative organizational model that merges various professionals, structures, and services transversally to make it more effective and homogeneous and coordinates the clinical path of patients with BC.

Recruitment will target women diagnosed with BC who are enrolled in the Breast Unit program, with additional support from local voluntary associations where possible. A convenience sampling approach will be used. Textbox 1 [14,17,28-30] outlines the study’s inclusion and exclusion criteria.

**Inclusion and exclusion criteria.**

**Inclusion criteria**

- Have a diagnosis of stage 0, I, II, or III breast cancer (BC)
- Be a female patient under the care of the Breast Unit
- Be aged ≥18 years (Italian legal age)
- Have a smartphone with internet access and the ability to download the app and be able to use it
- Be a resident of the Autonomous Province of Trento
- Know and understand the Italian language
- Have a moderate level of psychological distress, defined by a score between 15.9 and 29.9 on the Kessler Psychological Distress Scale, with higher scores indicating more severe distress [28]. This range aligns with the inclusion thresholds used in World Health Organization (WHO) studies using the RESPOND protocol [17].
- Have a general well-being score of ≥3 on the General Health Questionnaire, consistent with the inclusion criterion used in World Health Organization studies using the Self Health Plus protocol [14].
- Obtain a score between 30 and 80 (cutoffs identified based on the literature on the Short Form Health Survey 36 [SF-36] instrument in patients diagnosed with BC [29,30]) on the “Mental health” scale of the SF-36 questionnaire.

**Exclusion criteria**

- Patients unable to provide informed consent (prerequisite for participation in the study)
- Inadequate understanding of the Italian language
- Patients aged >75 years
- Women with metastatic BC (stage IV), those in the metastatic phase, receiving palliative care, or with a life expectancy of <1 year
- Obtain a score <30 on the “Bodily Pain” scale of the SF-36, indicating severe pain levels [29,30]
- Suicidal tendencies, depression, or other psychiatric diagnoses
- Already receiving psychological or psychotherapeutic care at the time of recruitment

Health professionals at the Breast Unit will verify the inclusion and exclusion criteria related to the sociodemographic data (phase 0), while psychologists will assess the psychological screening data (phase 1).

Participants will be required to sign an informed consent form during recruitment (phase 0), following a comprehensive explanation of the project, its objectives, data collection, management, processing procedures, the required level of involvement, the study duration, and confidentiality matters. They will be informed that they can withdraw from the study at any time without providing a reason and that doing so will not affect the quality of their care or interfere with their treatment. Informed consent must be given freely and in writing, accompanied by a data processing authorization form, before joining the study. Participants must provide written informed consent. They will also receive a privacy policy statement explaining how their personal data will be processed during the study and must provide explicit consent for its use. Copies of the informed consent and privacy forms will be provided to participants and available in a dedicated section of the app.

To promote participant engagement and retention throughout the 6-week intervention, specific features were designed to sustain interest and minimize dropout, such as incorporating gamification elements, providing regular feedback or progress tracking, scheduling personalized reminders, and offering small recognition for continued participation. No monetary compensation was provided.

#### Sample Size

This research project is designed as a POC study, where a limited sample is sufficient to achieve the intended aims. On the basis of calculation assuming a nonnormal distribution and using nonparametric statistics, a sample of 24 women with BC diagnoses is sufficient when applying a Bonferroni-corrected significance level (α=.025) to control for type I error and multiple comparisons. Nevertheless, to validate this estimate, the sample calculation was run with the Power Analysis and Sample Size 2021 (NCSS, LLC) and Stata (StataCorp LLC) programs. The resulting sample size is 41 participants, based on the following assumptions: a significance level (α) of .025, statistical power of 0.80 (to ensure sufficient sensitivity to type 2 error), a mean difference between groups (eg, pre- and postintervention questionnaires) of 1.00, an expected effect size (δ) of 0.50, and an SD of 2.00.

The chosen effect size (Cohen *d*=0.50) reflects a moderate change [31], which is consistent with previous digital mental health interventions in populations with psychological disorders [32]. This approach aligns with recommended practices in early-stage mHealth research, where the focus is on identifying potential signals of efficacy rather than definitive effect estimates [22]. Therefore, considering a 20% possible dropout [33], 50 women were estimated to need to be recruited to carry out the study. This approach is also coherent with previous studies adopting this type of methodology in the field of mHealth.

### Study Outcomes

#### Primary Outcomes

The primary outcomes are UX and usability. They will be assessed using 4 questionnaires, namely, the User Engagement Scale-Short Form (UES-SF) [34], the System Usability Scale (SUS) [35], the Italian Chatbot Usability Scale, version B (ITA BUS B) [36], and the User Mobile Application Rating Scale (uMARS) [37]. They will be administered at different time slots throughout the study, in particular, at the end of week 3 (UES-SF, SUS, and ITA BUS B) and at the end of the study (week 6: UES-SF, SUS,; ITA BUS B, and uMARS; Textbox 2).

**Summary of the questionnaires administered and their timing.**

**Screening**

- Kessler Psychological Distress Scale (K10)
- General Health Questionnaire (GHQ)
- Short Form Health Survey 36 (SF-36)

**At the beginning of the study (week 0)**

- Perceived Stress Scale (PSS)
- Multidimensional Psychological Flexibility Inventory (MPFI)
- General Self-Efficacy Scale (GSE)
- Emotion Regulation Questionnaire (ERQ)
- Coping Orientation to Problems Experienced‐New Italian Version 25 (COPE-NVI-25)

**At the end of week 3**

- User Engagement Scale-Short Form (UES-SF)
- System Usability Scale (SUS)
- Italian Chatbot Usability Scale, version B (ITA BUS B)

**At the end of the study (week 6)**

- GHQ
- PSS
- MPFI
- GSE
- ERQ
- COPE-NVI-25
- UES-SF (this will be administered the following day so as not to burden the participant during completion)
- SUS (this will be administered the following day so as not to burden the participant during completion)
- ITA BUS B (this will be administered the following day so as not to burden the participant during completion)
- User Mobile Application Rating Scale (uMARS; this will be administered the following day so as not to burden the participant during completion)

**Follow-up (4 months after the conclusion)**

- PSS
- Qualitative interview

The end points to be measured include the scores of individual questionnaire items, the average scores of the questionnaires, and the differences between average scores of the questionnaires at various survey times. In addition, the participants’ experiences with the app and interactions with ALBA, as well as their use of the intervention, will be evaluated through semistructured interviews conducted 4 months after the study concludes. These interviews will collect qualitative data to supplement the primary outcome information.

#### Secondary Outcomes

The variables expressing the psychological well-being outcomes will be assessed by administering self-report questionnaires at the beginning (screening and week 0) and at the end (week 6) of the intervention (Textbox 2 provides a detailed overview of the questionnaire adopted). The end points to be measured include the scores of individual questionnaire items, the average scores of the questionnaires, and the differences between the average scores of the questionnaires at 2 survey times. In addition, the Perceived Stress Scale (PSS) [38] will be administered during a follow-up conducted 4 months after the study’s conclusion.

Both the clinical and usability tests will yield within-subject and between-subject comparison results.

### Data Collection

The data collection process will take place in all the phases of the research, starting with recruitment and ending with follow-up.

#### Phase 0: Recruitment

Sociodemographic data collection will be carried out by the health professionals at the Breast Unit (physicians and case manager nurses) using a prestructured form. After informed consent is obtained, each form will be assigned a unique alphanumeric code. The parameters requested from participants will be (1) date of birth, (2) oncological disease history (ie, date of first cancer diagnosis, date of tumor removal surgery, date of any other interventions carried out or planned, and date of last neoadjuvant or adjuvant chemotherapy or radiotherapy treatment or if still ongoing), (3) maximum education grade, (4) employment, (5) marital status, and (6) current or previous experience of psychological support (exclusion criterion 7 verification).

#### Phase 1: Screening

To fulfill the verification of the inclusion criteria from 7 to 9 and the exclusion criteria 5 and 6, the researchers will administer the screening questionnaires through LimeSurvey (LimeSurvey GmbH; Textbox 2).

After processing the results of the screening questionnaires and the various sociodemographic inclusion criteria, the women will be contacted again, and the following scenarios will open.

First, the participant does not meet the inclusion criteria due to demonstrating a markedly positive state of psychological well-being, as indicated by a stress level <15.9 on the Kessler Psychological Distress Scale (K-10) [28], a general well-being score <3 on the General Health Questionnaire (GHQ) [39], and a general well-being score >80 on the “Mental Health” scale of the Short Form Health Survey 36 (SF-36) [29]. In such cases, the women will not be enrolled in the study, and the reasons for exclusion will be shared along with the screening questionnaire results, but upon the participant’s request.

Second, the participant does not meet the inclusion criteria due to exhibiting a markedly negative state of psychological well-being or signs of psychopathology, as indicated by a score <30 on mental health scale of the SF-36 and a K-10 score >29.9. In addition, in the second case, the participant will not be able to be enrolled in the study, and the reasons for exclusion from the study will be returned, and only upon request of the participant will the results obtained from the screening questionnaires be returned. Furthermore, indications of local services to contact for problems will be appropriately provided. This is to provide the participant with a structured and agreed system of referral to a qualified support service and taking charge where appropriate.

Third, the participant meets the inclusion criteria. In this case, the participant is enrolled in the study. The participant will also be asked if they would like to participate in a qualitative interview and in compiling a last questionnaire about stress 4 months after delivery. In case of positive feedback, the telephone number will be collected to facilitate contacts and follow-up interviews.

#### Phases 2 and 3: Enrollment and Participation

##### Overview

To properly assess included participants’ experience during the psychoeducational pathway, some questionnaires were adopted and delivered at the beginning (phase 2), during, and at the end of the interaction with ALBA (phase 3), as shown in Textbox 2.

Specifically, 5 questionnaires will be administered at the beginning (week 0) and at the end of the intervention (week 6) together with the initial screening questionnaires, which aim to investigate stress level, self-efficacy, emotional regulation, psychological flexibility, coping strategies, well-being, and level of psychological well-being. None of these questionnaires has diagnostic purposes. Therefore, they will not be used to diagnose psychopathology but only to collect descriptive data.

Four more questionnaires will be sent during the process to analyze the usability and involvement of the participant with the app and with the virtual coach, ALBA. Specifically, the usability and UX questionnaires will be administered at the end of week 3 (3 questionnaires) and week 6 (4 questionnaires).

A detailed description of each instrument that will be administered is presented in the subsequent sections.

##### UX and Usability Evaluation

The ITA BUS B [36] is a tool designed to evaluate the ease of use, effectiveness, and user satisfaction in interacting with the chatbot. While it is commonly used with artificial intelligence (AI)–powered chatbots, it is also applicable to other types of chatbot systems [36,40,41]. The 11 items are rated on a 5-point Likert scale, where 1 means “strongly disagree” and 5 means “strongly agree,” resulting in a total score ranging from 11 to 55. The areas investigated are as follows: perceived accessibility to chatbot functions, perceived quality of chatbot, perceived quality of conversation and information provided, perceived privacy and security, and time response.

SUS [35] is a quick and reliable tool for evaluating the usability of systems. Consisting of 10 items with Likert-type answers from 1 (“strongly agree”) to 5 (“strongly disagree”), the questionnaire provides a global score that reflects the ease of use and applicability of a system or product. The SUS yields a single number representing a composite measure of the overall usability of the system being studied, with a global score that can range from 0 to 100, depending on the degree of agreement with sentences such as “I found the system very cumbersome to use.”

UES-SF [34] measures user engagement with digital technology. Consisting of 12 items, the scale offers a quick but practical assessment of user engagement. Each item is rated on a 5-point Likert scale ranging from 1 (“strongly disagree”) to 5 (“strongly agree”), yielding a total score between 12 and 60. The questionnaire consists of four factors: (1) focused attention, which indicates the feeling of being immersed in the interaction; (2) perceived usability, which is the negative effect experienced due to the interaction and the effort expended; (3) aesthetic attractiveness, which represents the graphical and visual appeal of a digital solution; and (4) the reinforcement factor (reward). The latter is a single factor that includes duration, which evaluates the overall success of the interaction; novelty, which examines the general interest related to the interaction with a digital solution; and the perceived engagement factor, which evaluates the overall enjoyment of the interaction. As the questionnaire was not available in Italian, it was translated using the back-translation procedure. The 4 scales show good internal reliability, as follows: focused attention (ω=0.82), perceived usability (ω=0.86), aesthetic attraction (ω=0.84), and reinforcement (ω=0.81).

uMARS [37] is a tool for evaluating the quality of mobile apps. It provides a 20-item measure that includes 4 objective quality subscales—engagement, functionality, esthetics, and information quality—and 1 subjective quality subscale. One further subscale, consisting of 6 items, is added to measure users’ perceived impact of the evaluated app. This allows participants to obtain an in-depth and multidimensional evaluation of the quality of mobile apps. Each item offers answers on a Likert scale from 1 to 5, with 1 corresponding to “insufficient” and 5 to “excellent.”

##### Psychological Assessment

The Coping Orientation to Problems Experienced‐New Italian Version 25 (COPE-NVI-25) [42] is a shortened version of the Coping Orientation to Problems Experienced Inventory, designed to evaluate different coping strategies individuals use to deal with stress. This 25-item questionnaire explores strategies such as active coping, planning, and acceptance, providing a complex picture of an individual’s coping behavior. Specifically, 5 dimensions are examined: problem orientation, transcendent orientation, positive attitudes, social support, and avoidance strategies. These dimensions are assessed using a Likert scale ranging from 1 (“I never do it”) to 4 (“I often do it”), resulting in a total score between 25 and 100.

The Emotion Regulation Questionnaire [43] is a tool that evaluates 2 main emotion regulation strategies: expressive suppression and cognitive reappraisal. The questionnaire includes 10 items, with responses on a Likert scale from 1 (“strongly disagree”) to 7 (“strongly agree”), and is used to understand how individuals control and modify their emotional reactions in various contexts. On the basis of the answers, the score can vary from 10 to 70. Balzarotti et al [44] translated and validated the questionnaire in Italian, showing good psychometric characteristics.

GHQ-12 [39] is a self-assessment tool designed to identify mental health disorders and to monitor general psychological well-being. Because of its brevity, GHQ-12 has become one of the most widely used tools for detecting psychological distress. GHQ is widely used in clinical, research, and public health settings. The 12 items of the questionnaire are formulated to investigate how the individual has felt recently concerning psychological symptoms (such as anxiety and depression), ability to cope with daily situations, sleep disorders, and somatic symptoms. The response is based on a 4-point Likert scale, ranging from “not at all” to “much more than usual,” with a maximum score of 36.

The General Self-Efficacy Scale (GSE) [45] is a psychometric tool designed to evaluate an individual’s general perception of self-efficacy, that is, confidence in organizing and executing the actions necessary to manage potentially stressful or difficult situations. The GSE consists of 10 statements on which respondents must express their level of agreement on a Likert scale ranging from 1 (“not at all true”) to 4 (“exactly true”). The scale items explore various aspects of self-efficacy, such as the ability to solve problems, overcome obstacles, and handle unexpected situations. The total score, obtained by adding the scores of all the items, reflects the general level of self-efficacy perceived by the individual and can vary from 10 to 40, with a higher score indicating more self-efficacy.

K-10 [28] is a widely used screening tool to measure psychological distress. The K-10 is a short questionnaire composed of 10 items that investigate the frequency of psychological symptoms experienced by the individual in the last month, such as nervousness, sadness, tiredness, and feelings of hopelessness, for example, “During the last 30 days, about how often did you feel tired out for no good reason?” Each question is rated on a 5-point scale, ranging from “none of the time” to “all of the time.” Total scores range from 10 to 50, with higher scores indicating greater psychological distress (scores ≥30 suggest a high likelihood of a severe mental disorder).

The Multidimensional Psychological Flexibility Inventory (MPFI) [46] is a tool designed to assess psychological flexibility across multiple dimensions. It investigates how people adapt their thoughts and behaviors in response to changing situations and challenges. The MPFI focuses on various aspects of psychological flexibility. In particular, there are 6 scales for flexibility (acceptance, present moment awareness, self as context, defusion, values, and committed action) and 6 scales for inflexibility (experiential avoidance, lack of contact with the present moment, self as content, fusion, lack of contact with values, and inaction), according to the Hexaflex model [46]. For each of these dimensions, there are 5 items with responses ranging from 1 (“never true”) to 6 (“always true”).

PSS is a self-report questionnaire used to measure perceived stress. In particular, the objective is to determine the degree to which situations are perceived as stressful in a person’s daily life. Using 10 items rated on a 5-point Likert scale, this tool assesses participants’ thoughts and feelings over the past month regarding specific events, such as how often they felt unable to control important aspects of their lives. For each item, participants indicate the frequency on a scale from 0 (“never”) to 4 (“very often”).

SF-36 [29] is the most widely used general health measurement tool. It is a questionnaire divided into 8 sections: physical functioning (10 items), limitations due to physical health (4 items), limitations due to emotional problems (3 items), energy and fatigue (4 items), emotional well-being (5 items), social activities (2 items), pain (2 items), and perception of general health (36 items). For each dimension, the scores of the questions are coded, summed, and transformed into a scale ranging from 0 (worst health state) to 100 (best health state).

The platform adopted for the intervention allows for the recording of activity logs, enabling proper mapping of patients’ actions and experiences. This covers a range of logs, assessing whether the participant is properly exposed to the intervention protocol while recording use patterns (eg, session execution time, number of accesses, and use patterns of different features). Included among the variables considered are any missed sessions or interruptions. These data will be correlated with acceptability scores during the analysis phase. If a patient does not complete activities and fails to fill in specific questionnaires (leading to missing data), specific information on patients’ experiences and platform use will be collected during the scheduled postintervention interview sessions.

#### Phase 4: Follow-Up

The follow-up interview will be carried out 4 months after the conclusion of the SH+ process; will last approximately 20 minutes; and, subject to the patient’s consent, will be audio-recorded to allow subsequent analysis. The interviews are structured with ad hoc items specifically designed to capture key characteristics of the study and participants’ experiences. They are conducted by psychologists from the design team. During the qualitative interview, the patient will also be asked if someone supported them during the intervention, how easy it was to carry out the exercises, and general personal perceptions about the usability and real-world application of this app. Interviews will be conducted 4 months after the end of the intervention and will last approximately 20 minutes.

### Privacy and Data Management

At the time of recruitment, privacy documentation is available in line with Articles 13 and 14 of EU Regulation 2016/679 (General Data Protection Regulation), with specific focus on purposes and legal grounds of data processing, including information on how the personal data will be collected, the types of personal data collected, how the data will be managed, the retention period, the responsibilities of data controllers, and the rights of data subjects. Personal data are processed for specific research purposes within the scope of public interest tasks of the data controllers. Informed, freely given, voluntary, and explicit consent regarding the processing of special personal data (ie, data concerning health and data on self-reported behavioral habits in the area of lifestyle health) will be collected. Moreover, specific consent will be collected on the possibility of contacting the data subject via telephone to conduct research interviews. All data collected will be kept confidential, and data necessary for evaluating the study objectives will be pseudoanonymized before processing.

Access to personal or sensitive data is strictly limited to authorized IT specialists solely for the purpose of platform maintenance and in accordance with standard operating procedures and data protection protocols. App developers and platform hosts do not access personal or sensitive user data for any other purpose.

Copies of the information on the study and of the privacy policy will be issued to the participant and made available in a dedicated section of the app.

The study manager produces a report on the research and ensures that the data are reported responsibly and consistently. Personal data will neither be disclosed nor disseminated, except in anonymized or aggregated form for publication purposes. The transmission or dissemination of the data will take place exclusively in anonymous form, using only aggregated data that prevent any direct or indirect identification of the participants.

### Data Analysis

#### Overview

Preliminary data analysis is scheduled for early November 2024, while the final results will be available at the end of the study. The publication of the results will take place within 1 year after the conclusion of the study. Given the potential for dropout, which is commonly observed in digital interventions, and the sensitive nature of our sample population, missing data will be carefully addressed during the analysis phase. Where appropriate, multiple imputation methods will be applied to account for missing values and reduce potential bias. In addition, participants who drop out or fail to complete the follow-up assessments will be included in the analysis using an intention-to-treat approach, wherever feasible.

#### Quantitative Analysis

Statistical data processing will be conducted using the software R (version 4.0.0; R Foundation for Statistical Computing) [47], SPSS (IBM Corp) statistics [48], Stata (version 17) [49], and Jeffreys’s Amazing Statistics Program [50].

Categorical variables will be summarized using absolute frequencies and percentage distributions, while quantitative variables will be summarized using specific measures of central tendency and variability. Descriptive analyses will cover psychological variables (stress level, anxiety level, depression level, emotional regulation, psychological flexibility, coping strategies, well-being, and health status) as well as UX and usability variables. Relationships between variables will be analyzed primarily using ad hoc statistical tests, including the chi-square test, Fisher exact test, paired *t* test, Wilcoxon signed rank test, and sign test, depending on the assumptions met. These tests will help identify differences between the beginning and end of the course within the same study sample for the variables under investigation. In addition, univariate logistic or multinomial regression models will be presented. To account for potential confounding factors, multiple regression models will be proposed, adjusting the effects of explanatory variables on outcome variables for possible confounders. Statistical significance will be found for each analysis with a *P* value ≤.05*.* The McNemar test [51] and Cochran Q test [52] will also be used to evaluate paired qualitative data.

#### Qualitative Analysis

A qualitative analysis will be conducted using a combination of text mining techniques and thematic analysis. With regard to the analysis of the final semistructured interviews, the text mining approach [53] will be used to extract the answers that appear repeatedly from the interviews that will be conducted with respect to the women’s experience of interacting with ALBA and with respect to how they felt during the study participation. In particular, frequencies will be analyzed by identifying recurring words or phrases and exploring the dominant lexicon. Classifying texts according to emotional tone (positive, negative, and neutral) will also allow us to analyze sentiment and polarity. This is very useful for analyzing feedback, reviews, and opinions [54]. The specific software or tools to support this analysis (eg, NVivo [55]; Lumivero) are currently being evaluated. The final selection will be based on their ability to efficiently code and interpret open-ended responses, ensuring alignment with the study’s objectives and methodological framework.

Finally, significant segments (quotes) will also be extracted, and then, all results will be interpreted in the light of the literature.

### Ethical Considerations

This study received approval from the ethics committee of the Provincial Health Services Authority of Trento (Azienda Provinciale per i Servizi Sanitari di Trento; A985; July 17, 2024). At recruitment, all women who voluntarily choose to participate will be asked to sign an informed consent form after receiving a thorough explanation of the study, its objectives, the level of involvement required, the duration of the research, and all ethical issues related to confidentiality. While the health professionals of the Breast Unit will provide part of the explanation, a video recorded by the research team will also be available, clearly detailing the study; its purpose; and the participants’ involvement in simple, understandable terms. The participants will be informed of the study’s results through the app. Data will be pseudonymized and used only in aggregate form for research purposes. Participation is voluntary, and no compensation will be provided.

## Results

The literature highlights the growing interest in ACT-based interventions due to their efficacy in reducing anxiety, depression, and fear of recurrence and enhancing psychological flexibility and quality of life in patients with cancer [56]. ACT techniques, such as mindfulness and cognitive defusion, help manage anxiety and depressive symptoms, improve emotional regulation, and facilitate better stress management due to acceptance principles. Moreover, we expect an enhanced quality of life because through value-based actions, users could improve their overall well-being and life satisfaction. Consequently, we intend to incorporate variables such as psychological flexibility (MPFI), self-efficacy (GSE), coping strategies (COPE-NVI-25), and emotion regulation (Emotion Regulation Questionnaire) as predictors to our results. These variables are crucial for understanding the association between the ACT protocol and health outcomes, ensuring a comprehensive analysis of ACT’s efficacy across different phases of cancer treatment.

From a technical standpoint, collaboration with computer science experts will continue throughout the study to ensure the app is properly designed and maintained. We referred to internal usability studies and the previous co-design paper [24] as key examples to evaluate and refine the app’s functionalities before this pilot phase, but all new technical issues, such as bugs and glitches, will be monitored online throughout the development of the chatbot. This study aimed to identify and address any technical usability challenges before proceeding with a larger-scale trial. In other words, the findings from this feasibility study will inform decisions about scaling up the intervention or proceeding to a larger efficacy trial. Fundamental benchmarks will be based on key outcomes such as participant engagement rates, retention and dropout rates, significant changes in psychological variables (as mentioned earlier), and outcomes related to the acceptability and usability of the ALBA app, as measured by self-report questionnaires, along with the app’s functionality, including bug tracking. If these benchmarks are met, consideration will be given to moving forward with a larger, more definitive trial to assess the intervention’s efficacy. In addition, potential modifications to the intervention or trial design will be discussed, based on the results of this feasibility study, ensuring that the next phase could be better tailored to the needs of the target population in terms of content, functionality, and delivery methods.

## Discussion

### Anticipated Findings and Comparison to Previous Work

This POC study’s results can provide valuable data about the use, acceptance, and efficacy of this psychoeducational support for patients with BC receiving care through the Breast Unit. Previous studies indicated that patients diagnosed with BC found this form of support well accepted [57,58], suggesting that digital interventions can help address barriers related to social stigma and help seeking in patients with cancer, while also effectively enhancing patients’ psychological well-being.

The app examined in this study features personalized health plans tailored to users’ unique needs, which can lead to improved adherence to self-help treatment protocol and better health outcomes, particularly in terms of reduced stress, anxiety, and depression levels. Furthermore, the capacity for progress monitoring allows for timely interventions, which are crucial for also managing chronic conditions effectively [59]. The psychoeducational resources integrated into our app could promote health literacy and patient engagement among individuals with BC, empowering them to actively participate in their health management [60]; this is achieved through the innovative, interactive, and technology-driven delivery of ACT techniques.

Indeed, ALBA, as a basic and easily deliverable tool, could be offered to patients with BC in cases of mild to moderate psychological distress within a stepped care framework. This approach positions the ALBA app as a form of psychological first aid that allows a more efficient allocation of clinical resources, such as specialized psycho-oncological support, prioritized for more severe or urgent cases [61]. In this way, the intervention can serve as a widely accessible and user-friendly option, particularly for patients who may be hesitant to seek traditional psychological support or who are placed on waiting lists due to the mild nature of their distress.

Summing up, mHealth apps, such as the one described in this paper, offer promising advantages, such as increased accessibility, scalability, cost-effectiveness, and potential to reduce stigma around mental health care [10-12]. However, they are also accompanied by significant challenges. Key concerns include data privacy and security, limited regulatory oversight, and variability in clinical effectiveness, especially in the absence of robust evidence and standardized evaluation methods [11,12,24,57]. Although these tools may support self-management and extend care to underserved populations, they often rely on users’ technological literacy and self-discipline, limiting their usability and adherence over time [10,62]. Tools, such as uMARS [37], have been developed to assess app quality, yet a persistent gap remains in validating content and ensuring sustained engagement. Moreover, overreliance on such interventions may reduce face-to-face interactions with health care professionals, potentially weakening the therapeutic alliance and care continuity. This concern is particularly relevant for populations considered vulnerable, including older adults and individuals with low digital literacy, who may experience reduced access or engagement [63]. Indeed, the digital divide remains a significant issue, as only some have access to or are proficient with digital technologies, which can exacerbate disparities in health care access [64]. Furthermore, issues such as reduced personalization, risk of self-diagnosis, and a lack of integration with traditional clinical pathways raise questions about their long-term safety [57,62]. Therefore, digital health solutions such as ALBA should be implemented as complementary to, rather than substitutes for, human-delivered care, ensuring that personal support and professional guidance remain accessible and central to the care pathway [62]. This aspect, including organizational implications of these technologies in terms of health care delivery, could be explored in future studies.

Finally, we plan to implement AI capabilities in ALBA in the future. The usability and UX questionnaires—particularly those used in this study, along with the psychological assessments—will play an important role in supporting the validation process and ensuring the system’s continuous improvement. It is important to note that ALBA and TreC research are not intended to replace clinical or medical support but rather to complement them by promoting psychological well-being and healthy lifestyles during BC care, including in cases where AI is integrated.

### Strengths, Limitations, and Future Directions

One of the strengths of this study protocol is the involvement of experts from various disciplines related to oncology and psycho-oncology, as well as experts of SH+ working for WHO, in the design phase. This multidisciplinary collaboration not only enhances the protection of the interests of patients with BC but also ensures a more comprehensive and well-rounded perspective in the codevelopment of the intervention [65]. Another major strength is integrating the study within a structured behavioral intervention design framework, specifically the ORBIT model [22]. This approach adds methodological rigor and scientific validity to the intervention’s development and the subsequent testing phases. Second, adhering to a standardized framework facilitates comparisons with previous literature, ensures replicability, and enables a more robust evaluation of the intervention’s effectiveness. Positioned after the co-design phase [24,65], this POC study provides an initial assessment of the app’s usability and potential efficacy. However, it does not explore how ACT-based psychoeducation affects other important issues for patients with BC, such as body image, sexual quality of life, chronic fatigue, or dyadic adjustment following surgery [66,67]. A limitation of this study is the absence of a control group, which limits the ability to draw comparative conclusions. Future research should consider more robust designs, including larger randomized controlled trials. Moreover, although age was accounted for in the inclusion criteria, we acknowledge that digital literacy is a distinct and equally relevant factor influencing participants’ ability to engage with digital mental health tools. Future studies will consider incorporating specific assessment tools, such as the eHealth Literacy Scale [68], to evaluate participants’ comfort with and proficiency in using digital technologies at the time of recruitment. Finally, the limitation of using self-report tests and the inherent lack of control, in our case, of the social desirability bias, is typical of psychological intervention protocols. Because this is a POC study with voluntary participation in a clinical setting, we believe, however, that if the study is replicated in the future as a clinical trial or expanded, it will be important to consider this factor. For instance, it could be useful to use response evaluation tools that focus on indirect evaluation methodologies, such as the Implicit Association Test [69], or scales specifically targeting social desirability, such as the Marlowe‐Crowne Social Desirability Scale [70]. Finally, within the broader framework of the ALBA study, a dissemination project is planned. This will cover both the Italian national and regional territories where the app will be tested, involving clinical stakeholders, hospitals, and support services. The plan also includes outreach to the wider scientific community through participation in conferences and publication in scientific journals, aiming to promote the use of digital mental health interventions, highlighting their strengths and weaknesses, and engaging sector experts in potential multicultural replications of our study.

### Conclusions

The preliminary results from the previous ALBA co-design study and results from the scientific literature suggest that delivering consistent psychoeducational CBT support via mHealth solutions may be both acceptable and potentially useful for women undergoing BC treatment. Therefore, the POC study’s findings illustrated in this protocol paper could contribute to the growing body of evidence supporting the use of digital technologies in mental health care. In particular, these findings could offer valuable insights into the usability, acceptance, and effectiveness digital mental health interventions in promoting psychological well-being among populations that face barriers and typically have limited access to psychological support.

## Abbreviations

ACT: acceptance and commitment therapy
AI: artificial intelligence
ALBA: A Well-Being Assistant
BC: breast cancer
CBT: cognitive behavioral therapy
COPE-NVI-25: Coping Orientation to Problems Experienced‐New Italian Version 25
GHQ: General Health Questionnaire
GSE: General Self-Efficacy Scale
ITA BUS B: Italian Chatbot Usability Scale, version B
K-10: Kessler Psychological Distress Scale
mHealth: mobile health
MPFI: Multidimensional Psychological Flexibility Inventory
ORBIT: Obesity-Related Behavioral Intervention Trials
POC: proof-of-concept
PSS: Perceived Stress Scale
RESPOND: preparedness of health systems to reduce mental health and psychosocial concerns resulting from the COVID-19 pandemic
SH+: Self-Help Plus
SF-36: Short Form Health Survey 36
SUS: System Usability Scale
TreC: cartella clinica del cittadino; (“citizen’s electronic health record”)
UES-SF: User Engagement Scale-Short Form
uMARS: User Mobile Application Rating Scale
UX: user experience
WHO: World Health Organization

## Data Availability

The datasets generated or analyzed during this study are available from the corresponding author upon reasonable request.
